# Influence of Cryopreservation of Pre-Implantation Embryos on the Epigenome

**DOI:** 10.3390/cells15121049

**Published:** 2026-06-08

**Authors:** Tom Trapphoff, Ursula Eichenlaub-Ritter, Karoline Hohenstein, Saskia Möckel, Stefan Dieterle

**Affiliations:** 1 Dortmund Fertility Center, 44135 Dortmund, Germany; hohenstein@kinderwunschzentrum.org (K.H.); moeckel@kinderwunschzentrum.org (S.M.); dieterle@kinderwunschzentrum.org (S.D.); 2 Department of Gene Technology/Microbiology, Faculty of Biology, University of Bielefeld, 33615 Bielefeld, Germany; eiri@uni-bielefeld.de; 3 Division of Reproductive Endocrinology and Infertility, Department of Obstetrics and Gynaecology, Witten/Herdecke University, 44135 Dortmund, Germany

**Keywords:** medically assisted reproduction, pre-implantation embryos, cryopreservation, epigenome, DNA methylation, post-translational histone modification, non-coding RNA

## Abstract

The cryopreservation of pre-implantation embryos has become routine in medically assisted reproduction (MAR), and the proportion of frozen embryo transfers has steadily increased in recent years. Because cryopreservation through either slow-cooling protocols or ultra-rapid vitrification requires potentially cytotoxic cryoprotective agents to prevent uncontrolled and detrimental ice crystal formation, the safety of these procedures must be carefully considered. Evidence from human epidemiological studies, including retrospective and prospective controlled studies, and data from national patient registries indicate that children born after frozen embryo transfer have a higher birth weight than those born after spontaneous conception and have an increased risk of rare genomic imprinting disorders, such as Beckwith–Wiedemann, Silver–Russell, or Prader–Willi syndrome. Encompassing not only reversible DNA methylation patterns established during gametogenesis, but also the timed abundance and availability of transcripts and proteins required to establish or maintain epigenetic marks throughout development and differentiation, as well as persistent or transient post-translational histone modifications and non-coding RNAs, the epigenome may be particularly sensitive to cryopreservation. Importantly, epigenetic regulation is highly complex. Alterations of the epigenome at any developmental stage are often not monocausal, do not necessarily result in immediate disturbances in the pre-implantation embryo, and are unlikely to operate through simple all-or-nothing mechanisms; however, they may have long-lasting effects at later developmental stages. To make matters even more complex, differences between species in terms of epigenetic regulation or lineage differentiation are well known and translation from animal model systems to humans must be considered with caution. More recently, epigenetic regulation by non-coding RNAs has also come into focus, as these molecules are crucial, either directly or indirectly, for gene expression, translation, and protein biosynthesis during development. Therefore, assessing potential adverse effects of cryopreservation on the entire epigenome remains a major challenge, particularly because little is known about indirect factors, such as post-translational histone modifications and non-coding RNAs. In this review, we focus on the potential influence of the cryopreservation of pre-implantation embryos on the epigenetic profile in humans and animals. Specifically, we consider DNA methylation of imprinted genes and global DNA methylation; post-translational histone modifications; the abundance and availability of transcripts and proteins required to establish, maintain, or protect epigenetic patterns; and the presence of non-coding RNAs involved in epigenetic control.

## 1. Introduction

Since the birth of Louise Brown in 1978 after the first successful in vitro fertilisation (IVF) in humans, medically assisted reproduction (MAR) has become a standard procedure for infertility treatment and fertility preservation [1,2]. Overall, more than 10 million babies have been born after MAR worldwide since 1978 [3]. MAR comprises a broad spectrum of techniques, including controlled ovarian stimulation and retrieval of mature oocytes, oocyte in vitro maturation or culture, conventional IVF or intracytoplasmic sperm injection (ICSI), and the transfer of fresh or cryopreserved zygotes or pre-implantation embryos at different stages of in vitro development. Since the first births after cryopreservation of pre-implantation embryos and oocytes, cryopreservation of immature and mature gametes, ovarian and testicular tissue, and zygotes and pre-implantation embryos has become routine practice. The demand for this approach has steadily increased in recent years, and the proportion of frozen embryo transfers (FETs) has continued to rise [4,5,6]. Accordingly, in recent years, an estimated 30% of all births after MAR have involved cryopreserved embryos, zygotes, or oocytes [6].

Two main cryopreservation procedures, with several variations, have been used in clinical and experimental settings: slow-cooling protocols and ultra-rapid vitrification techniques. Both methods require the exposure of tissues or cells to cryoprotective agents (CPAs), which commonly include ethylene glycol (EG), glycerol, propylene glycol (PG), propanediol (PrOH), and dimethyl sulfoxide (DMSO), to prevent uncontrolled and detrimental ice crystal formation [7,8]. Non-permeating agents, such as sugars, proteins, and other macromolecules, are also used to achieve controlled cell dehydration and cellular CPA uptake. Ultra-rapid vitrification protocols typically involve high CPA concentrations and short exposure times to achieve an amorphous, glass-like solidification state after gametes or embryos are plunged directly into liquid nitrogen [8]. Slow-cooling protocols, by contrast, use lower CPA concentrations combined with longer exposure times and induce small ice crystals by seeding during a controlled freezing process [7]. Different CPAs are known to have toxic effects on cellular functions and structures; for instance, exposure to DMSO can lead to alterations of the epigenome in different cell types, including misregulated expression of DNA methyltransferases, DNA hypo- or hypermethylation, and alterations of post-translational histone modifications [9,10,11]. Thus, vitrification, and especially residual CPAs after vitrification/warming, might pose a higher risk of cytotoxic CPA exposure compared with slow-freezing protocols due to the higher CPA concentrations needed for vitrification, even though shorter exposure times are used [12]. However, both methods have been used successfully. Whereas slow-freezing protocols are recommended for ovarian tissue preservation [13], vitrification has become the “gold standard” for the preservation of metaphase II oocytes, zygotes, and pre-implantation embryos in medically assisted reproduction [14].

Because MAR may affect the integrity of gametes and embryos, particularly through exposure to potentially toxic CPAs during cryopreservation [9,10,11], the general safety of MAR procedures has been analysed in a large number of epidemiological and both retrospective and prospective controlled studies since early reports in 2009 [3,15,16,17,18]. Overall, the risk to the integrity of gametes and embryos and to the health of children born after MAR, including cryopreservation, appears to be low [3,18], and advanced maternal age is one of the major risk factors for adverse outcomes [19,20]. However, differences have been reported between offspring conceived spontaneously and those born after fresh or cryopreserved MAR cycles. For example, children born after fresh embryo transfer have higher rates of preterm birth and lower birth weight, whereas infants born after frozen embryo transfer have higher birth weights than those born after spontaneous conception [3,18]. Moreover, children conceived after MAR, with or without cryopreservation, have a slightly increased risk of childhood malignancies, cardiometabolic disorders, congenital abnormalities, and rare genomic imprinting disorders, such as Beckwith–Wiedemann syndrome (BWS), Silver–Russell syndrome (SRS), and Prader–Willi syndrome (PWS) [18,21,22,23,24,25,26]. Long-term follow-up data from human clinical cohorts has also indicated metabolic and neurodevelopmental disorders linked to epigenetic alterations in offspring; for instance, children conceived through MAR had a lower level of fasting insulin, higher level of fasting glucose, and abnormal lipid metabolism compared to children conceived naturally. Additionally, an increased risk of neurodevelopmental disorders such as intellectual disability, delayed cognitive development, and cerebral palsy have been reported, although many of the identified risk associations were not present after adjustment [16,18,27]. To date, it remains unclear whether these slight increases in adverse outcomes are related to MAR itself or instead reflect intrinsic factors in the subfertile patient population undergoing MAR. In particular, inherited disturbances in the epigenetic landscape of gametes, embryos, and offspring have been discussed as a potential contributor to increased health risks in children born after MAR, including cryopreservation [18].

**Figure 1:**
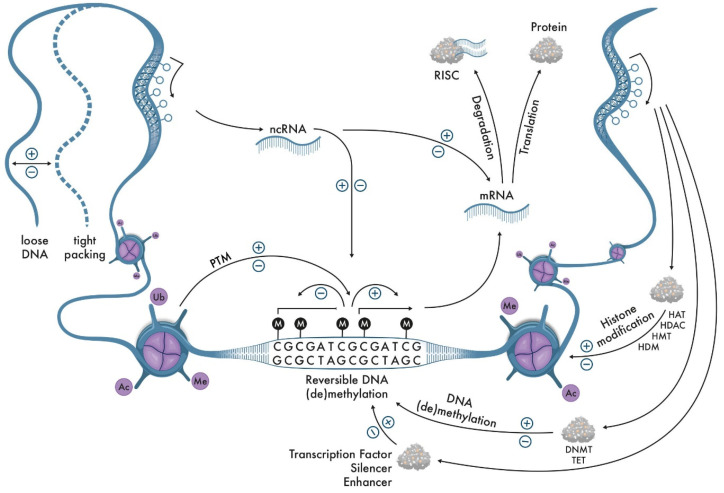
Schematic overview of epigenetic regulation by reversible DNA methylation and demethylation, post-translational histone modifications (PTMs), and non-coding RNAs (ncRNAs). DNA methylation and demethylation are mediated by DNA methyltransferases (DNMTs) and ten-eleven translocation (TET) methylcytosine dioxygenases, respectively. Histone modifications, including methylation (Me), acetylation (Ac), and ubiquitination (Ub), are regulated by histone acetyltransferases (HATs), histone deacetylases (HDACs), histone methyltransferases (HMTs), and histone demethylases (HDMs). The epigenetic network also includes ncRNAs, which can regulate transcription, mRNA stability, transcript degradation by the RNA-induced silencing complex (RISC), and translation.

The epigenome can be regarded as a superimposed, metastable state of cells and tissues that controls spatiotemporal gene expression without altering the nucleobase sequence itself. The epigenetic landscape includes reversible DNA base modifications, such as methylation and demethylation; post-translational histone modifications (PTMs), including methylation, acetylation, phosphorylation, and ubiquitination; the abundance and availability of transcripts and proteins required to establish or maintain epigenetic marks; and, as has increasingly come into focus in recent years, non-coding RNAs (ncRNAs) (Figure 1) [28,29,30,31]. Specific epigenetic modifications are transmitted transgenerationally or are differentially erased and re-established in a highly controlled, development-dependent, and tissue-specific manner. Such modifications are therefore not rigid, but are instead subject to dynamic reprogramming during gametogenesis, fertilisation, embryogenesis, and differentiation, thereby ensuring cell-autonomous, differentiation-specific, and tissue-specific gene expression patterns [32,33,34]. Epigenomic signatures can be modified according to metabolic demands and may be severely disturbed, for instance in response to exogenous and endogenous stressors, ageing, lifestyle, or disease [35,36].

**Figure 2:**
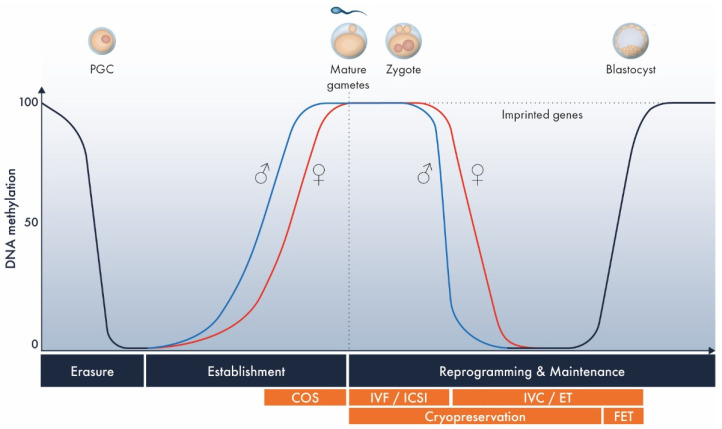
DNA methylation dynamics during gametogenesis and early embryogenesis. DNA methylation patterns are erased in primordial germ cells (PGCs) followed by the de novo establishment of parental-specific DNA methylation profiles in male and female gametes. After fertilisation, global DNA demethylation occurs during early embryogenesis, whereas methylation marks at imprinted genes are maintained. Cell- and tissue-specific de novo DNA methylation is subsequently established during embryogenesis and differentiation. Orange bars indicate the timing of MAR procedures relative to stage-specific epigenetic reprogramming: controlled ovarian stimulation (COS), in vitro fertilisation (IVF)/intracytoplasmic sperm injection (ICSI), in vitro culture (IVC), embryo transfer (ET), cryopreservation, and frozen embryo transfer (FET). Cryopreservation is either performed with mature metaphase II oocytes (after establishment of parental-specific DNA methylation profile) or mature or immature spermatozoa, zygotes, and cleavage-stage embryos, or at the blastocyst stage (during reprogramming and maintenance of the epigenetic profile that occurs in early embryonic development). Scheme adopted from Trapphoff et al. [43].

DNA methylation at distinct CpG (cytosine–phosphate–guanine dinucleotide) sites and post-translational modification of histone amino acid residues within chromatin represent key elements of the epigenetic landscape that contribute to the activation or repression of gene expression. The somatic DNA methylation pattern is erased during gametogenesis in primordial germ cells, and gamete- and sex-specific DNA methylation profiles in female and male gametes are then established de novo (Figure 2). In post-pubertal females, global DNA methylation (gDNA) is established in follicle-enclosed oocytes before maturation is resumed in meiotically arrested growing prophase I oocytes prior to ovulation [33]. In males, gamete-specific DNA methylation takes place during meiosis at the pachytene stage of spermatogenesis including the replacement of histones with protamines to induce crucial sperm DNA compaction [32]. During the early divisions of the pre-implantation embryo, passive demethylation occurs in the maternally inherited genome during replication, except at imprinted sites that are essential for controlling the expression of imprinted genes at later developmental stages and in adulthood [34,37,38,39]. By contrast, the paternally inherited genome undergoes active global DNA demethylation during chromatin remodelling after fertilisation by ten-eleven translocation (TET) methylcytosine dioxygenases, except at paternally imprinted regions. Importantly, the sex-specific maternally or paternally inherited DNA marks at imprinted genes are essential for development and monoallelic gene expression in the embryo and adult and must be retained in zygotic DNA, whereas cell- and tissue-specific de novo DNA reprogramming is established during embryogenesis and differentiation (Figure 2). The de novo establishment of parent-specific DNA methylation at imprinted genes occurs during gametogenesis in sperm and oocytes at specific CpG sites within differentially methylated regions (DMRs). Several of the 200 known imprinted genes are organised in imprinted control regions (ICRs) [37,38]. Loss of DNA methylation in specific DMRs of imprinted genes is associated with imprinting disorders, such as Beckwith–Wiedemann, Prader–Willi, and Silver–Russell syndrome [40,41,42].

To ensure proper and stage-specific DNA methylation, the abundance and availability of transcripts and proteins, including the de novo methyltransferases DNMT3A and/or DNMT3B/DNMT3L, maintenance DNA methyltransferase 1 (DNMT1), several ten-eleven translocation (TET) methylcytosine dioxygenases, zinc finger protein 57 (ZFP57), and MATER protein homologue (MATER), are required to protect imprinted genes from demethylation and are crucial for correct DNA methylation and demethylation [44,45]. In addition to DNA methylation and its corresponding enzymatic network, specific post-translational histone modifications can directly or indirectly activate or repress gene expression through changes in chromatin conformation or by recruiting transcriptional, enhancer, or silencing factors [31,34]. To establish the most common PTMs, histone deacetylases (HDACs), histone acetyltransferases (HATs), histone methyltransferases (HMTs), and histone demethylases (HDMs) add or remove acetyl or methyl groups at specific histone residues [46]. Finally, ncRNAs, such as long non-coding RNAs (lncRNAs) and microRNAs (miRNAs), can regulate transcription or translation via cis- or trans-acting mechanisms to control gene expression. The modes of action of regulatory ncRNAs are diverse and include mRNA guidance, stabilisation, or degradation; initiation or repression of gene transcription; and regulation of PTMs [47,48].

Overall, the epigenetic regulation of gene expression is embedded in a dense and complex network. Alterations of the epigenome are often not immediately monocausal and do not rely on simple all-or-nothing mechanisms; thus, they may have long-lasting and potential cascading effects at later developmental stages, whereby even small alterations on the molecular level may have substantial consequences later on with regard to phenotype expression or, conversely, no consequences at all. Therefore, assessing and classifying potential adverse effects of MAR techniques on the epigenetic landscape remains a major challenge, particularly after cryopreservation. To make assessment even more complex, differences between species in terms of lineage differentiation, transcription, and epigenetic mechanisms are well known, such as those regarding the timing of zygotic gene activation during early embryogenesis, CpG density, histone modifications for transcriptional regulation, abundance and regulation of ncRNAs, and the epigenetic reprogramming mechanism during early embryonic development [49,50,51,52]. So far, several concerns regarding epimutations have been raised from animal studies in mice and bovines, showing that findings cannot been adopted in a simple way from animals to humans and that caution is absolutely necessary when translating results from animal model systems to human clinical implications. The influence of cryopreservation of immature gametes, ovarian and testicular tissue, and mature MII oocytes on the epigenetic profile has already been reviewed previously [43,53]. In this narrative review, we focus on the potential influence of the cryopreservation of pre-implantation embryos on the epigenetic profile in humans and animals. Specifically, we consider DNA methylation of imprinted genes and global DNA methylation, post-translational histone modifications, the abundance and availability of relevant transcripts and proteins, and the presence of ncRNAs.

## 2. Influence of Cryopreservation on Global DNA Methylation and Methylation of Transposable Elements

The global DNA methylation pattern and the methylation of transposable elements (TEs), such as long interspersed nuclear element-1 (LINE-1) and human endogenous retrovirus-FRD envelope protein (HERV-FRD), play essential roles in the epigenetic regulation of transcription in mature oocytes and early embryos. These specific marks are crucial for fertilisation, the oocyte-to-embryo transition, early development, placentation, and foetal growth [54,55,56]. Alterations of global DNA or TE methylation, potentially induced by cryopreservation, may therefore have detrimental and long-lasting effects.

Several studies have analysed gDNA and TE methylation levels and did not identify significant alterations after vitrification of pre-implantation embryos compared with non-vitrified controls (overview in Table 1). Zhu et al. [57], using single-cell whole-genome bisulphite sequencing (scWGBS), and Marjonen and colleagues [58], using EpiTYPER and bisulphite sequencing, reported similar gDNA and TE methylation patterns in human eight-cell embryos and placental tissue, respectively, after vitrification. In contrast, global DNA methylation was not altered after vitrification of murine two-cell embryos compared with non-vitrified in vitro controls but was decreased in the inner cell mass of blastocysts compared with in vivo controls, as assessed by immunocytochemistry [59].

Using bisulphite pyrosequencing, Barberet and colleagues [55] also showed no adverse effect on the degree of TE methylation in cord blood and placentas after frozen embryo transfer compared with natural conception in humans. However, LINE-1 methylation was decreased after fresh embryo transfer compared with natural conception and frozen embryo transfer. In mice, Rhon-Calderon et al. [60] analysed global DNA methylation patterns in foetuses and placentas after blastocyst vitrification and found no differences in methylation levels compared with non-vitrified controls, using a luminometric methylation assay (LUMA) and bisulphite sequencing.

However, several authors have reported hypermethylation, rather than hypomethylation, in humans and animals following cryopreservation at different developmental stages. Mani et al. [61] analysed placentas from vitrified human and murine blastocysts and showed increased DNA methylation after vitrification compared with non-vitrified in vitro controls, using MethylationEPIC BeadChip arrays and Mouse Methylation BeadChip arrays for analysis. Ying and Zhang [62] also described hypermethylation in blastocysts in mice after vitrification of eight-cell embryos compared with non-vitrified in vitro controls, using WGBS and immunofluorescence staining. Using LUMA, Gosh and colleagues [63] analysed global DNA methylation and the LINE-1 methylation profile in human placentas after cryopreservation of blastocysts and zygotes by slow-cooling techniques. Increased DNA methylation levels were observed after cryopreservation compared with non-vitrified controls and naturally conceived pregnancies. Consistent with the findings of Barberet and colleagues [55], LINE-1 methylation was increased after cryopreservation compared with non-vitrified in vitro controls but was not different from that in naturally conceived pregnancies.

Ma et al. [64] vitrified murine eight-cell embryos and analysed gDNA in foetuses and placentas using the MethylFlash Global DNA Methylation Kit, reporting that gDNA methylation was increased after vitrification and warming compared with non-vitrified in vitro controls. Interestingly, although methylation levels in placentas were significantly reduced compared with in vivo controls, gDNA methylation in foetuses did not differ significantly from that in the corresponding in vivo controls.

Using immunocytochemistry, Yao [65] found that global methylation marks significantly decreased in vitrified murine and bovine eight-cell embryos and corresponding blastocysts compared with in vivo and non-vitrified in vitro controls. Similar results were reported by Shida et al. [66] after vitrification of bovine eight-cell embryos using different CPAs. In immunocytochemical analyses, global DNA methylation was decreased after vitrification with DMSO or propylene glycol in eight-cell embryos and in blastocysts in the DMSO group, whereas it was increased in the propylene glycol group. In a study by Movahed [67], murine two-cell embryos were vitrified, and gDNA methylation was analysed in the corresponding blastocysts by immunocytochemistry, once again showing a decrease after vitrification compared with non-vitrified in vivo and in vitro controls.

In summary, findings regarding disturbances in global DNA and TE methylation are equivocal. Some studies reported no adverse effects in mice and humans after vitrification of cleavage-stage embryos or blastocysts in corresponding pre-implantation embryos, foetuses, and placental tissues when using ethylene glycol and DMSO as cryoprotective agents. However, some reported increased methylation patterns, mainly in foetuses and placentas, after vitrification of cleavage-stage embryos or blastocysts in mice and humans, when using the same cryoprotective agents. By contrast, other studies showed hypomethylation after vitrification, mainly between the eight-cell and blastocyst stages in mice and bovines. Thus, loss of methylation patterns at pre-implantation stages and compensatory or excessive remethylation at later stages may be one possible mechanism underlying these alterations as shown in animal model systems. In humans, hypomethylation was not demonstrated after cryopreservation in pre-implantation embryos or at later stages. Overall, when focusing in particular on clinical implications, the cryopreservation of pre-implantation embryos has a possible effect on global DNA and TE methylation. It is noteworthy that in vitro treatment itself, even without cryopreservation, may also have detrimental effects on the epigenetic landscape, as shown by several studies reviewed recently [27,68,69].

**Table 1:** Global DNA methylation levels. Cryopreservation was performed by vitrification unless stated otherwise. All CPA concentrations are in volume per volume (*v*/*v*); immunofluorescence (IF); luminometric methylation assay (LUMA); single-cell whole-genome bisulphite sequencing (scWGBS); equilibration solution (ES); vitrification solution (VS); ethylene glycol (EG); dimethyl sulfoxide (DMSO); propylene glycol (PG); not available (n.a.).

Reference	Year	Species	Cryopreserved Cell Type/Cryopreservation Protocol	Analysed Cell Type	Technique	Target	Finding
Bakhtari et al. [59]	2014	Mouse	2-cell embryos ES: 7.5% EG + 7.5% DMSO, 3 min VS: 15% EG + 15% DMSO, 45 s	Blastocysts	IF	Global DNA methylation	No differences compared with non-vitrified in vitro controls. Decreased global DNA methylation in inner cell mass after vitrification compared with in vivo controls.
Barberet et al. [55]	2021	Human	FET vs. fresh embryo transfer vs. natural conception No details available regarding cryopreservation protocol	Placenta and cord blood	Bisulphite pyrosequencing	DNA methylation profile of two transposable elements (LINE-1 and HERV-FRD)	No adverse effect on the degree of methylation in cord blood. In placentas, LINE-1 methylation was increased compared with fresh embryo transfers, but similar to that for natural conception.
Ghosh et al. [63]	2017	Human	Blastocysts and D1 zygotes (both slow-cooling) No details available regarding slow cooling protocol	Placentas	LUMA	Global DNA methylation and LINE-1 methylation profile	Increased global DNA methylation after cryopreservation compared with non-vitrified controls and naturally conceived pregnancies. Increased LINE-1 methylation after cryopreservation compared with in vitro controls, but no difference compared with naturally conceived pregnancies.
Ma et al. [64]	2019	Mouse	8-cell embryos ES: 7.5% EG + 7.5% DMSO, 5 min VS: 15% EG + 15% DMSO, 60 s	Foetuses and placentas	MethylFlash Global DNA Methylation Kit	Global DNA methylation	Increased global DNA methylation in foetuses and placentas after vitrification compared with in vitro controls. In foetuses, global DNA methylation was comparable to that in in vivo controls after vitrification. In placentas, global DNA methylation was reduced after vitrification compared with in vivo controls.
Mani et al. [61]	2022	Human/Mouse	Blastocysts Mouse: ES: 7.5% EG + 7.5% DMSO, 5 min VS: 15% EG + 15% DMSO, <60 s Human: data n.a.	Placentas	MethylationEPIC BeadChip array	Global DNA methylation	Vitrification led to placental DNA hypermethylation in mice and humans compared with non-vitrified in vitro controls.
Marjonen et al. [58]	2018	Human	FET vs. fresh embryo transfer vs. natural conception No details available regarding cryopreservation protocol	Placental tissue	EpiTYPER and bisulphite sequencing	DNA methylation levels of long interspersed nuclear elements	DNA methylation profiles of LINE-1 were not significantly different after frozen embryo transfer compared with non-vitrified controls and natural conception.
Movahed et al. [67]	2019	Mouse	2-cell embryos ES: 7.5% EG + 7.5% DMSO, 3 min VS: 15% EG + 15% DMSO, 60 s	Blastocysts	IF	Global DNA methylation	Decreased global DNA methylation after vitrification compared with non-vitrified in vivo and in vitro controls.
Rhon-Calderon et al. [60]	2024	Mouse	Blastocysts ES: 7.5% EG + 7.5% DMSO, 5 min VS: 15% EG + 15% DMSO, <60 s	Foetuses and placentas	LUMA and bisulphite sequencing	Global DNA methylation	Global DNA methylation did not differ from that in in vitro controls.
Shida et al. [66]	2025	Bovine	8-cell embryos ES: 7.5% EG + 7.5% DMSO or 7.5% PG, 5 min VS: 15% EG + 15% DMSO or 15% PG, 30 s	8-cell embryos/blastocysts	IF	Global DNA methylation	Decreased global DNA methylation after vitrification with DMSO in 8-cell embryos and blastocysts compared with non-vitrified controls. Using propylene glycol for vitrification, global DNA methylation was decreased at the 8-cell stage and increased in blastocysts compared with non-vitrified controls.
Yao et al. [65]	2017	Mouse	8-cell embryos ES: 7.5% EG + 7.5% DMSO, 2 min VS: 15% EG + 15% DMSO, 50 s	8-cell embryos/blastocysts	IF	Global DNA methylation	Reduced global DNA methylation in 8-cell embryos/blastocyst after vitrification compared with in vitro and/or in vivo controls.
Ying and Zhang [62]	2023	Mouse	8-cell embryos No details available regarding cryopreservation protocol	Blastocysts	WGBS and IF	Global DNA methylation	Increased global DNA methylation after vitrification compared with non-vitrified in vitro controls.
Zhu et al. [57]	2024	Human	8-cell embryos (mid- and long-term storage in LN_2_ for 3 and 8 years vs. non-vitrified controls) ES: 7.5% EG + 7.5% DMSO, 12–15 min VS: 15% EG + 15% DMSO, 45–60 s	8-cell embryos	scWGBS	Global DNA methylation including differentiation between functional genomic regions	No adverse effect on the overall degree of methylation compared with non-vitrified controls. Similar methylation patterns in functional regions among all groups.

## 3. Influence of Cryopreservation on Methylation Patterns of Imprinted and Pluripotency Genes

**Figure 3:**
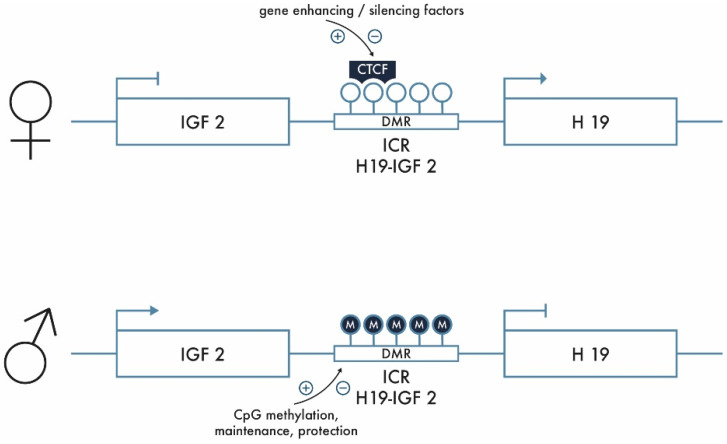
Schematic representation of the *H19*-*IGF2* imprinting control region (ICR) on maternally and paternally inherited alleles, ensuring monoallelic gene expression in the embryo and adult. On the maternal allele, the unmethylated differentially methylated region (DMR) allows for binding of CCCTC-binding factor (CTCF), which contributes to *H19* expression and *IGF2* repression. On the paternal allele, CpG methylation of the DMR prevents CTCF binding, supports maintenance and protection of the methylation pattern, permits *IGF2* expression, and represses *H19*.

To ensure parent-of-origin-specific gene expression after fertilisation, genomic imprinting occurs differentially in DMRs in male and female gametes. DMRs consist of multiple individual CpG methylation sites and are located within imprinting control regions (ICRs). Imprinted genes are often arranged in imprinted gene clusters, and a single ICR can regulate several imprinted genes (Figure 3) [70]. Although CpG methylation is the key mechanism underlying genomic imprinting, other molecules, such as CCCTC-binding factor (CTCF) and ncRNAs, including the lncRNA transcribed from the imprinted *KCNQ1OT1* gene itself, are also involved in monoallelic gene expression [71,72,73]. Genomic imprinting therefore depends mainly on CpG methylation, although aberrant hypermethylation or hypomethylation of individual CpGs does not always lead to altered gene expression. Thus, the interpretation of individual CpG epimutations after MAR treatment is complex and must be approached with caution.

Several authors found no epimutations after cryopreservation in humans or animals at different developmental stages (overview in Table 2). Derakhshan-Horeh and colleagues [74] used bisulphite sequencing to analyse *H19* and *IGF2* CpG methylation levels in human blastocysts after vitrification of day-3 embryos and found no adverse effects on the degree of methylation between non-vitrified controls and the vitrification group. Using EpiTYPER and bisulphite sequencing for CpG methylation analysis, Marjonen and colleagues [58] also found no differences in methylation patterns at the *H19* ICR and *H19* DMR in placental tissue in humans after frozen embryo transfer compared with fresh embryo transfer and natural conception. To assess the effects of single and double revitrification of human blastocysts, Movahedin et al. [75] analysed methylation levels of the *H19*/*IGF2* DMR in corresponding blastocysts using bisulphite sequencing, finding that *H19*/*IGF2* DMR methylation patterns did not differ after vitrification or revitrification compared with non-vitrified in vitro controls.

Yao et al. [76] analysed *SNRPN* DNA methylation by pyrosequencing in neonatal placental tissue after vitrification of human pre-implantation embryos, reporting no adverse effect on the degree of *SNRPN* methylation compared with non-vitrified in vitro and in vivo controls. The CpG methylation profile of the imprinted gene *Grb10* was analysed by Yao and colleagues [65] in blastocysts after vitrification of murine eight-cell embryos using bisulphite sequencing. No differences were found between vitrified and non-vitrified pre-implantation embryos, but *Grb10* CpG methylation was decreased after vitrification compared with in vivo controls. Similar results were reported by Hiura et al. [77] in human placentas using bisulphite restriction analysis. Methylation levels of the IG-DMR and C19MC-DMR were not significantly different after frozen embryo transfer, fresh embryo transfer, and natural conception. In addition, *MEG3*-DMR methylation was similar between FET and fresh ET but increased compared with natural conception. Although *OCT4* does not belong to the group of imprinted genes, it is important for establishing genomic pluripotency during early embryogenesis. In a study by Saenz de Juano [78], rabbit morulae were vitrified, and the methylation pattern of *OCT4* was analysed in the corresponding blastocysts using bisulphite sequencing. No significant differences in *OCT4* methylation levels were found after vitrification compared with non-vitrified controls.

Hypermethylation of imprinted genes has also been reported by several authors after vitrification. Hosseini et al. [79] analysed the methylation status of the promoter region of the imprinted gene *Meg3* in the hippocampus of D2 offspring after vitrification of murine two-cell embryos. Using methylation-specific PCR, they found higher *Meg3* methylation levels after vitrification compared with non-vitrified in vitro and in vivo controls. When analysing bovine two-cell embryos and corresponding blastocysts using bisulphite sequencing, Zhao et al. [80] found increased *H19* ICR methylation after vitrification at the two-cell stage compared with non-vitrified controls. In a study by Barberet and colleagues [55], the DNA methylation profiles of *H19*/*IGF2*, *KCNQ1OT1*, and *SNURF* were analysed in human placenta and cord blood by bisulphite pyrosequencing. In cord blood, no differences were present after FET compared with natural conception and fresh embryo transfer; by contrast, the DNA methylation level of *H19*/*IGF2*-seq2 was higher after frozen embryo transfer than after natural conception and fresh embryo transfer in placental tissue. Interestingly, as noted above, in vitro treatment, even without cryopreservation, may also lead to imprinting disorders. DNA methylation of *H19*/*IGF2*-seq1 was significantly lower in the fresh embryo transfer group than in the control group, whereas vitrification did not induce epimutations at this DMR.

In a study by Zhu and colleagues [57], the effect of mid- and long-term storage (3 vs. 8 years) of vitrified human eight-cell embryos in LN2 was analysed by scWGBS. Although methylation patterns of the 22 analysed imprinted genes did not differ after mid- or long-term storage compared with non-vitrified controls, hypermethylation of *DIRAS3* was shown after vitrification compared with non-vitrified controls at the eight-cell stage.

Ma et al. [64] analysed methylation levels of the imprinted gene *KvDMR1* in murine foetuses and placentas after vitrification of eight-cell embryos using bisulphite pyrosequencing. In placentas, methylation levels did not differ after vitrification compared with non-vitrified in vitro controls but were decreased compared with in vivo controls. In foetuses, *KvDMR1* hypermethylation was present after eight-cell embryo vitrification compared with non-vitrified in vitro controls. However, methylation levels were comparable to those in appropriate in vivo controls, supporting the assumption that in vitro treatment itself may induce epimutations.

By contrast, decreased methylation levels of imprinted genes, and of some pluripotency genes, have been reported after cryopreservation of pre-implantation embryos in humans and mice. Rhon-Calderon [60] analysed the methylation profiles of the imprinted regions *H19*/*Igf2*, IgDMR, *Peg3*, *Kcnq1ot1*, and *Snrpn* in foetuses and placentas after vitrification of murine blastocysts. Although this work focused on the effect of embryo biopsy, hypomethylation of Snrpn and IgDMR was shown after vitrification compared with appropriate in vitro controls in placentas and foetuses. In the latter, lower *H19*/*Igf2* DNA methylation was also shown after vitrification compared with non-vitrified controls using LUMA and bisulphite sequencing.

Wang [81] also analysed methylation levels of the *H19*/*Igf2* DMR in murine foetuses and placentas after morula vitrification using bisulphite sequencing. *H19*/*Igf2* methylation was reduced in foetuses after vitrification compared with in vivo and in vitro controls, and in placentas after vitrification and in vitro culture compared with in vivo controls.

In a study by Wu et al. [82], human foetal tissue was analysed by NGS-based bisulphite PCR after multifetal pregnancy reduction, and hypomethylation of *H19* promoter DNA was reported after cryopreservation of pre-implantation embryos obtained after IVF, ICSI, and FET compared with fresh embryo transfers. In addition, Zhao and colleagues [83] analysed the methylation status of four pluripotency and differentiation genes (*Oct4*, *Nanog*, *Cdx2*, and *Hand1*) after blastocyst vitrification in corresponding murine blastocysts. Using bisulphite sequencing, methylation levels of the *Oct4*, *Nanog*, and *Cdx2* promoters were decreased after vitrification compared with non-vitrified controls, whereas *Hand1* promoter methylation was not significantly different after vitrification.

Overall, the results for methylation patterns of imprinted genes and some relevant pluripotency genes are also controversial. Irrespective of the vitrified or analysed cell type and developmental stage, hypermethylation, hypomethylation, and similar methylation patterns have all been reported. In humans, several studies showed no adverse effects following vitrification of pre-implantation embryos—neither in the corresponding embryos themselves nor at later developmental stages—although some studies also showed higher DNA methylation of *H19*/*IGF2*-seq2 or *Diras3* in placental tissue or eight-cell embryos, or reduced *H19* promoter DNA methylation in foetal tissue. Overall, cryopreservation of pre-implantation embryos has a possible effect on methylation patterns of imprinted genes in humans and animal model systems. Thus, cryopreservation, but also in vitro treatment without cryopreservation, may induce epimutations at the CpG level.

**Table 2:** DNA methylation levels of imprinted genes. Cryopreservation was performed by vitrification unless stated otherwise. All CPA concentrations are in volume per volume (*v*/*v*); luminometric methylation assay (LUMA); single-cell whole-genome bisulphite sequencing (scWGBS); equilibration solution (ES); vitrification solution (VS); ethylene glycol (EG); dimethyl sulfoxide (DMSO); not available (n.a.).

Reference	Year	Species	Cryopreserved Cell Type/Cryopreservation Protocol	Analysed Cell Type	Technique	Target	Finding
Barberet et al. [55]	2021	Human	FET vs. fresh embryo transfer vs. natural conception Details regarding cryopreservation protocol n.a.	Placenta and cord blood	Bisulphite pyrosequencing	DNA methylation profile of three imprinted genes (*H19*/*IGF2*, *KCNQ1OT1*, *SNURF*)	In placental tissue, the DNA methylation level of *H19*/*IGF2*-seq2 was higher after FET compared with natural conception and fresh embryo transfer. In cord blood, no differences were observed among groups.
Derakhshan-Horeh et al. [74]	2016	Human	Day-3 embryos ES: 7.5% EG + 7.5% DMSO VS: 15% EG + 15% DMSO	Blastocysts	Bisulphite sequencing	*H19* and *IGF2* CpG methylation levels	No adverse effect on the degree of methylation after vitrification compared with non-vitrified controls.
Hiura et al. [77]	2017	Human	FET vs. fresh embryo transfer vs. natural conception ES: 7.5% EG + 7.5% DMSO VS: 15% EG + 15% DMSO	Placenta	Bisulphite restriction analysis	DNA methylation analysis of imprinted DMRs (IG-DMR and *MEG3*-DMR/C19MC-DMR)	Methylation levels of IG-DMR and C19MC-DMR were not significantly different among groups. *MEG3*-DMR methylation was similar between FET and fresh ET, but increased compared with natural conception.
Hosseini et al. [79]	2025	Mouse	2-cell embryos ES: 7.5% EG + 7.5% DMSO; 5 min VS: 15% EG + 15% DMSO; 30 s	Hippocampus of D2 offspring	Methylation-specific PCR	Methylation status at the promoter region of the imprinted gene *Meg3*	Higher methylation levels of *Meg3* after vitrification compared with in vitro and in vivo controls.
Ma et al. [64]	2019	Mouse	8-cell embryos ES: 7.5% EG + 7.5% DMSO, 5 min VS: 15% EG + 15% DMSO, 60 s	Foetuses and placentas	Bisulphite pyrosequencing	Methylation levels of imprinted gene *KvDMR1*	Methylation levels of *KvDMR1* were increased in foetuses after vitrification compared with non-vitrified in vitro controls and similar to those in in vivo controls. In placentas, methylation levels were decreased after vitrification compared with in vivo controls and similar to those in non-vitrified in vitro controls.
Marjonen et al. [58]	2018	Human	FET vs. fresh embryo transfer vs. natural conception Details regarding cryopreservation protocol n.a.	Placental tissue	EpiTYPER and bisulphite sequencing	DNA methylation levels at the *H19* ICR and *H19* DMR	DNA methylation profiles at the *H19* ICR and *H19* DMR were not significantly different after frozen embryo transfer compared with non-vitrified controls and natural conception.
Movahedin et al. [75]	2022	Human	Blastocysts (vitrified and revitrified) ES: 7.5% EG + 7.5% DMSO VS: 15% EG + 15% DMSO	Blastocysts	Bisulphite sequencing	Methylation levels of *H19*/*IGF2* DMR	Methylation levels of *H19*/*IGF2* DMR were not different after vitrification or revitrification compared with non-vitrified in vitro controls.
Rhon-Calderon et al. [60]	2024	Mouse	Blastocysts ES: 7.5% EG + 7.5% DMSO, 5 min VS: 15% EG + 15% DMSO, <60 s	Foetuses and placentas	LUMA and bisulphite sequencing	Methylation profile of the imprinted regions *H19*/*Igf2*, IgDMR, *Peg3*, *Kcnq1ot1*, and *Snrpn*	Decreased DNA methylation for *Snrpn* and IgDMR after vitrification compared with in vitro controls in placentas and foetuses. In foetuses, lower *H19*/*Igf2* DNA methylation was also observed after vitrification compared with non-vitrified controls.
Saenz de Juano et al. [78]	2014	Rabbit	Morulae ES: 12.5% EG + 12.5% DMSO, 2 min VS: 20% EG + 20% DMSO, 30 s	Blastocysts	Bisulphite sequencing	Methylation levels of pluripotency gene *OCT4*	No significant differences in *OCT4* methylation levels.
Wang et al. [81]	2010	Mouse	Morulae ES: 7.5% EG + 7.5% DMSO VS: 15% EG + 15% DMSO	Foetuses and placentas	Bisulphite sequencing	*H19*/*Igf2* differentially methylated domain	Loss of *H19*/*Igf2* methylation was observed in foetuses after vitrification compared with in vivo and in vitro controls, and in placentas after vitrification and in vitro culture compared with in vivo controls.
Wu et al. [82]	2024	Human	Cryopreserved IVF/ICSI embryos vs. fresh IVF cycles No details available regarding cryopreservation protocol	Foetal tissue after multifetal pregnancy reduction	NGS-based bisulphite PCR	Methylation levels of the *H19* promoter and *H19* imprinting control element	Reduced *H19* promoter DNA methylation after cryopreservation of embryos obtained after IVF and ICSI compared with fresh IVF embryos.
Yao et al. [65]	2017	Mouse	8-cell embryos ES: 7.5% EG + 7.5% DMSO, 2 min VS: 15% EG + 15% DMSO, 50 s	Blastocysts	Bisulphite sequencing	CpG methylation profile of imprinted gene *Grb10*	No differences compared with non-vitrified in vitro blastocysts. Decreased *Grb10* CpG methylation after vitrification compared with in vivo controls.
Yao et al. [76]	2020	Human	FET vs. fresh embryo transfer vs. natural conception No details available regarding cryopreservation protocol	Neonatal placental tissue	Pyrosequencing	*SNRPN* DNA methylation	No adverse effect on the degree of methylation.
Zhao et al. [83]	2012	Mouse	Blastocysts EG- and DMSO-based open-pulled straw vitrification protocol	Blastocysts	Bisulphite sequencing	Methylation status of four pluripotency and differentiation genes (*Oct4*, *Nanog*, *Cdx2*, *Hand1*)	Reduced methylation levels of the *Oct4*, *Nanog*, and *Cdx2* promoters after vitrification compared with non-vitrified controls. *Hand1* promoter methylation was not significantly different after vitrification.
Zhao et al. [80]	2012	Bovine	2-cell embryos ES: 10% EG + 10% DMSO, 30 s VS: 15% EG + 15% DMSO, 25 s	2-cell embryos/blastocysts	Bisulphite sequencing	Methylation levels of the *H19* imprinted control region	Increased *H19* methylation after vitrification at 2-cell stage in 2-cell embryos and corresponding blastocysts compared with non-vitrified controls.
Zhu et al. [57]	2024	Human	8-cell embryos (mid- and long-term storage in LN_2_ for 3 and 8 years vs. non-vitrified controls) ES: 7.5% EG + 7.5% DMSO, 12–15 min VS: 15% EG + 15% DMSO, 45–60 s	8-cell embryos	scWGBS	DNA methylation of 23 imprinted DMRs	DMR methylation did not differ between mid- and long-term storage, but *Diras3* methylation was higher compared with non-vitrified controls.

## 4. Influence of Cryopreservation on Gene Expression of Imprinted Genes

Whether, and to what extent, altered CpG methylation after cryopreservation results in altered gene expression is of great importance (overview in Table 3). Zhu et al. [57] analysed the gene expression profile of 23 imprinted genes after vitrification of human eight-cell embryos using single-cell RNA-seq, and found no significant differences at the eight-cell stage between vitrified and non-vitrified pre-implantation embryos, not even for the imprinted gene *DIRAS3*, for which higher CpG methylation levels were found after vitrification. Movahedin and colleagues [75] also found no differences in the expression levels of *H19* and *IGF2* in humans after single vitrification or revitrification of blastocysts compared with non-vitrified controls, as assessed by qRT-PCR; thus, DNA methylation levels of the *H19*/*IGF2* DMR also did not differ.

Without providing data on CpG methylation patterns, Yodrug et al. [84] analysed bovine blastocysts after vitrification by qRT-PCR, finding that expression levels of *IGF2R* and *SNRPN* did not differ after vitrification compared with non-vitrified blastocysts. Similar results were reported by Dliyaul Haq [85], Jahangiri [86,87], and Movahed [88] in mice. In the first study [85], the mRNA content of *H19* and *Igf2* did not differ significantly between morulae and blastocysts with or without vitrification, as assessed by qRT-PCR. Jahangiri and colleagues [86] analysed the expression levels of the imprinted genes *H19* and *Mest* in blastocysts after two-cell-stage vitrification and subsequent in vitro culture by qRT-PCR, finding no differences between vitrified and non-vitrified in vitro controls. However, both expression levels were increased after vitrification compared with fresh in vivo controls. In a follow-up study by the same authors with a similar experimental design [87], no differences in *Igf2* expression were shown between vitrified and non-vitrified blastocysts; however, in both groups, expression levels were upregulated compared with in vivo controls. Comparable results were reported by Movahed [88]. After vitrification of two-cell embryos and in vitro culture to the blastocyst stage, no differences in *Dlk1* gene expression were shown between vitrified and non-vitrified in vitro controls at the blastocyst stage, whereas *Dlk1* expression was increased compared with in vivo controls. qRT-PCR was used for gene expression analysis. For these latter studies in mice, no information on DNA methylation patterns was available.

Because gene transcription does not necessarily result in protein expression, the study by Yao et al. [76] is of particular interest. *SNRPN* mRNA levels were analysed by qRT-PCR, and protein content was assessed by Western blotting in human neonatal placental tissue after vitrification of pre-implantation embryos. No differences in gene or protein expression were observed after fresh embryo transfer or FET in placental tissue; however, similarly to findings in mice [86,88], increased mRNA and protein levels were shown after fresh or frozen embryo transfer compared with natural conception.

In contrast to studies showing no differences between vitrified and non-vitrified in vitro controls, several authors reported altered gene expression profiles after cryopreservation. In bovine embryos, Zhao and colleagues [80] vitrified two-cell cleavage-stage embryos and analysed *H19* expression levels at the blastocyst stage by qRT-PCR. Coinciding with an altered *H19* CpG methylation profile, *H19* mRNA was downregulated after vitrification compared with non-vitrified controls. Using qRT-PCR for analysis after vitrification of murine eight-cell embryos and in vitro culture to the blastocyst stage, Yao et al. [65] showed decreased *Grb10* expression after vitrification compared with non-vitrified in vitro and in vivo controls, even in the absence of alterations in the CpG methylation profile of *Grb10*. Similar results were also reported in mice by Sahraei and colleagues [89]: *H19* and *Igf2* expression was downregulated after vitrification of eight-cell embryos and in vitro culture to the blastocyst stage compared with non-vitrified controls, whereas *Mest* expression was unaffected. qRT-PCR was used for gene expression profiling, but no information on CpG methylation levels was available.

Barberet et al. [55] analysed the transcription profiles of three imprinted genes (*H19*, *KCNQ1*, and *SNRPN*) in human placentas and cord blood after frozen embryo transfer, fresh embryo transfer, and natural conception. In cord blood, no differences in gene expression were observed among groups using qRT-PCR; in placenta, *H19* was downregulated after vitrification of pre-implantation embryos compared with non-vitrified in vitro controls. However, the *H19* expression profile was comparable to that in natural pregnancies. Interestingly, the DNA methylation level of *H19*/*IGF2*-seq2 was higher after FET than after natural conception and fresh embryo transfer in placenta, whereas no differences were present among groups in cord blood.

Another study of particular interest was published by Bartolac et al. [90], in which the authors analysed not only the potential influence of vitrification, but also CPA exposure (EG/DMSO) alone without subsequent vitrification, on porcine blastocysts. Expression levels of the imprinted genes *IGF2* and *IGF2R* were analysed by qRT-PCR at the blastocyst stage: whereas *IGF2* and *IGF2R* mRNA levels after CPA exposure without vitrification were similar to those in non-vitrified control blastocysts, *IGF2* and *IGF2R* were downregulated after the complete vitrification process compared with non-vitrified controls. Thus, vitrification, but not CPA exposure alone, may induce epimutations. However, it should be noted that DMSO exposure without subsequent cryopreservation may still induce alterations of the epigenetic landscape in different in vitro cell culture types or animal embryos, as shown by several authors [9,10,11,91,92].

Upregulation of gene expression after vitrification was shown in mice by Hosseini et al. [79] for the imprinted gene *Igf2* compared with non-vitrified in vitro and in vivo controls. Two-cell embryos were vitrified, and expression of *Meg3*, *Snrpn*, *H19*, and *Igf2* was analysed in the hippocampus of D2 offspring by qRT-PCR. Expression levels of *Snrpn* and *H19* were unaffected. Moreover, *Meg3* expression was lower after vitrification compared with in vivo controls, but not compared with non-vitrified in vitro controls. Interestingly, higher CpG methylation levels of the *Meg3* promoter region were shown after vitrification compared with both controls, supporting the assumption that altered CpG methylation can, but does not necessarily, lead to gene expression defects. In this context, Wang et al. [81] vitrified murine morulae and analysed the expression levels of *H19* and *Igf2* in placentas and foetuses by qRT-PCR, showing upregulation of *H19* and decreased *Igf2* expression in placentas and foetuses after vitrification compared with non-vitrified in vitro and in vivo controls. Interestingly, methylation patterns of the *H19*/*Igf2* DMR were also altered in this study. In contrast to the work of Hosseini and colleagues [79], dysregulation of ICR CpG methylation in the latter study may have directly affected gene expression.

**Table 3:** Expression levels of imprinted genes. Cryopreservation was performed by vitrification unless stated otherwise. All CPA concentrations are in volume per volume (*v*/*v*); equilibration solution (ES); vitrification solution (VS); ethylene glycol (EG); dimethyl sulfoxide (DMSO); propanediol (PrOH); not available (n.a.).

Reference	Year	Species	Cryopreserved Cell Type/Cryopreservation Protocol	Analysed Cell Type	Technique	Target	Finding
Barberet et al. [55]	2021	Human	FET vs. fresh embryo transfer vs. natural conception No details available regarding cryopreservation protocol	Placenta and cord blood	qRT-PCR	Transcription profile of three imprinted genes (*H19*, *KCNQ1*, *SNRPN*)	Decreased *H19* expression in placenta after frozen embryo transfer compared with fresh embryo transfer.
Bartolac et al. [90]	2018	Porcine	Blastocysts ES: 7.5% EG + 7.5% DMSO or 7.5% PrOH, 3 min VS: 17% EG + 17% DMSO or 17% PrOH, 30–45 s	Blastocysts	qRT-PCR	Expression levels of imprinted genes *IGF2* and *IGF2R*	Decreased expression of *IGF2* and *IGF2R* compared with non-vitrified controls.
Dliyaul Haq et al. [85]	2019	Mouse	Morulae/blastocysts ES: 10% EG VS: 15% EG + 15% DMSO	Morulae/blastocysts	qRT-PCR	Expression levels of imprinted genes *H19* and *Igf2*	No differences in *H19* and *Igf2* mRNA content after morulae and blastocyst vitrification compared with in vivo controls.
Hosseini et al. [79]	2025	Mouse	2-cell embryos ES: 7.5% EG + 7.5% DMSO; 5 min VS: 15% EG + 15% DMSO; 30 s	Hippocampus of D2 offspring	qRT-PCR	Gene expression levels of imprinted genes (*Meg3*, *Snrpn*, *H19*, *Igf2*)	Upregulation of *Igf2* after vitrification compared with in vivo and non-vitrified controls. No differences in *Meg3* expression compared with non-vitrified in vitro controls, but decreased expression after vitrification compared with in vivo controls.
Jahangiri et al. [86]	2014	Mouse	2-cell embryos ES: 7.5% EG + 7.5% DMSO; 7 min VS: 15% EG + 15% DMSO; 60 s	Blastocysts	qRT-PCR	Expression levels of imprinted genes *H19* and *Mest*	No differences compared with non-vitrified in vitro blastocysts. Increased expression levels of *H19* and *Mest* after vitrification compared with in vivo controls.
Jahangiri et al. [87]	2018	Mouse	2-cell embryos ES: 7.5% EG + 7.5% DMSO; 7 min VS: 15% EG + 15% DMSO; 60 s	Blastocysts	qRT-PCR	Expression levels of imprinted gene *Igf2*	No differences between vitrified and non-vitrified blastocysts, but increased expression levels of *Igf2* in both groups compared with in vivo controls.
Ma et al. [64]	2019	Mouse	8-cell embryos ES: 7.5% EG + 7.5% DMSO, 5 min VS: 15% EG + 15% DMSO, 60 s	Foetuses and placentas	qRT-PCR	Expression levels of 24 imprinted genes (*Dlk1*, *Igf2*, *Kcnq1ot1*, *Mest*, *Ndn*, *Peg3*, *Plagl1*, *Sgce*, *Snrpn*, *Cd81*, *Cdkn1c*, *Dcn*, *Gatm*, *Gnas*, *Grb10*, *Gtl2*, *H19*, *Igf2r*, *Mash2*, *Osbpl5*, *Phlda2*, *Slc22a18*, *Ube3a*, *Zim1*)	Altered gene expression of several imprinted genes (e.g., *Sgce*, *Dcn*, *Gtl2*, *Kcnq1ot1*) in foetuses/placentas after vitrification compared with in vivo and/or non-vitrified in vitro controls. Several maternally expressed genes were upregulated, which may have repressed foetal growth.
Movahed et al. [88]	2020	Mouse	2-cell embryos ES: 7.5% EG + 7.5% DMSO; 3 min VS: 15% EG + 15% DMSO; <60 s	Blastocysts	qRT-PCR	Expressions levels of imprinted gene *Dlk1*	No differences compared with non-vitrified in vitro controls. Increased mRNA levels of *Dlk1* after vitrification compared with in vivo controls.
Movahedin et al. [75]	2022	Human	Blastocysts (vitrified and revitrified) ES: 7.5% EG + 7.5% DMSO VS: 15% EG + 15% DMSO	Blastocysts	qRT-PCR	Expression levels of imprinted genes *H19* and *IGF2*	Expression levels of *H19* and *IGF2* were not different after vitrification or revitrification compared with non-vitrified in vitro controls.
Sahraei et al. [89]	2018	Mouse	8-cell embryos Details regarding cryopreservation protocol n.a.	Blastocysts	qRT-PCR	Expression levels of imprinted genes *H19*, *Igf2*, and *Mest*	Downregulation of *H19* and *Igf2* after vitrification compared with non-vitrified controls. *Mest* expression was unaffected.
Wang et al. [81]	2010	Mouse	Morulae ES: 7.5% EG + 7.5% DMSO VS: 15% EG + 15% DMSO	Foetuses and placentas	qRT-PCR	Expression levels of imprinted genes *H19* and *Igf2*	Upregulation of *H19* and decreased *Igf2* expression in placentas and foetuses compared to in vitro and in vivo controls
Yao et al. [76]	2020	Human	FET vs. fresh embryo transfer vs. natural conception No details available regarding cryopreservation protocol	Neonatal placental tissue	qRT-PCR and Western blot	Expression of *Snrpn* mRNA and SNRPN protein	No differences in gene or protein expression after fresh or frozen embryo transfer. Increased levels of mRNA and protein after fresh or frozen embryo transfer compared to natural conception.
Yao et al. [65]	2017	Mouse	8-cell embryos ES: 7.5% EG + 7.5% DMSO, 2 min VS: 15% EG + 15% DMSO, 50 s	Blastocysts	qRT-PCR	Expression levels of imprinted gene *Grb10*	Decreased expression level of *Grb10* after vitrification compared to non-vitrified in vitro blastocysts and in vivo controls.
Yodrug et al. [84]	2020	Bovine	Blastocysts ES: 2% or 7.5% EG + 2% or 7.5% DMSO, 3 min VS: 16.5% EG + 16.5% DMSO, 50 s	Blastocysts	qRT-PCR	Expression levels of two imprinted genes (*IGFR2*, *SNRPN*)	Expression of *IGF2R* and *SNRPN* not different after vitrification compared to non-vitrified controls.
Zhao et al. [80]	2012	Bovine	2-cell embryos ES: 10% EG + 10% DMSO, 30 s VS: 15% EG + 15% DMSO, 25 s	Blastocysts	qRT-PCR	Expression levels of imprinted gene *H19*	Decreased *H19* mRNA expression after vitrification compared to non-vitrified controls.
Zhu et al. [57]	2024	Human	8-cell embryos (mid- and long-term storage in LN_2_ for 3 and 8 years vs. non-vitrified controls) ES: 7.5% EG + 7.5% DMSO, 12–15 min VS: 15% EG + 15% DMSO, 45–60 s	8-cell embryos	Single-cell RNA-seq	Gene expression of 23 imprinted genes	Expression levels of 23 imprinted genes did not differ after vitrification compared with non-vitrified controls.

The largest dataset to date on gene expression profiles of imprinted genes was reported by Ma et al. [64]. Overall, 24 paternally or maternally expressed imprinted genes were analysed by qRT-PCR in placentas and foetuses after vitrification of murine eight-cell embryos. Several genes were upregulated (e.g., *Kcnq1ot1* and *Gtl2*) or downregulated (e.g., *Dcn* and *Sgce*) in placentas or foetuses after vitrification compared with non-vitrified in vitro and in vivo controls. Nevertheless, in vitro culture alone also affected the expression of several imprinted genes in placentas and foetuses (e.g., *Gatm* and *Cd8*). Another important aspect of this study was the assessment of foetal growth in vitrified, non-vitrified, and naturally conceived foetuses: foetal crown–rump length at E9.5 was significantly reduced after in vitro culture with or without vitrification compared with the naturally conceived group. With respect to gene expression profiles, most maternally expressed genes were upregulated after vitrification or in vitro culture alone, which could repress foetal growth.

Overall, findings regarding the expression profiles of imprinted genes after cryopreservation of pre-implantation embryos are inconsistent. Although several authors found no alterations, many reported upregulation or downregulation of imprinted genes. No clear relationship could be established between CpG methylation levels, where available, and gene expression profiles; therefore, other upstream or downstream mechanisms are likely to influence gene expression. The study by Ma et al. [64] is of particular interest with respect to MAR outcomes in humans. As mentioned earlier, children born after fresh embryo transfer have lower birth weights, whereas infants born after frozen embryo transfer have higher birth weights than those born after spontaneous conception [3]. There is a well-known duality between maternally and paternally expressed imprinted genes in the control of foetal and postnatal development: the former are often involved in limiting resource flow to the foetus, whereas the latter promote it [93]. Alterations induced by MAR treatment, with or without cryopreservation, may disturb parent-of-origin-specific gene expression during embryogenesis, which could explain differences in birth weight after medically assisted reproduction. Thus, cryopreservation of pre-implantation embryos has a possible effect on the expression profiles of imprinted genes in humans and animal model systems.

## 5. Influence of Cryopreservation on PTM Profiles

Epigenetic marks established through methylation, acetylation, or ubiquitination of histone residues regulate chromatin packaging and accessibility, thereby permitting gene transcription or repression. For instance, H3K4me3 is commonly associated with gene expression and cell differentiation, whereas H3K9me3 is associated with heterochromatin [31,34,94,95,96,97,98,99]. Post-translational histone modifications occur in a stage-specific manner and are essential for gene activation and silencing during oogenesis, spermatogenesis, and early embryogenesis [31,34].

Bakhtari et al. [59] vitrified murine two-cell embryos and analysed the status of histone methylation (H3K4me3) and acetylation (H3K9ac and H4K12ac) at the blastocyst stage by immunostaining. No differences in H3K9ac, H4K12ac, or H3K4me3 were found compared with non-vitrified in vitro controls; however, H4K12ac acetylation was increased, and H3K4me3 methylation was decreased after vitrification compared with in vivo controls.

No differences between vitrified and non-vitrified embryos in post-translational histone modifications for H3K9ac, H3K9me2, and H3K4me3 were reported by Jahangiri et al. in two studies [86,87], in which murine two-cell embryos were vitrified, and histone modification profiles were analysed. Using sensitive chromatin immunoprecipitation (ChIP) assays, the authors analysed specific PTMs at CTCF-binding sites of the imprinted *H19*/*Igf2* gene region and the *Mest* promoter region, respectively. In contrast to the findings of Bakhtari et al. [59], H3K4me3 methylation levels did not differ after in vitro culture with or without vitrification compared with in vivo controls. However, the H3K9ac profile was increased and H3K9me2 was decreased in both groups compared with in vivo controls.

In bovine embryos, Souza Cáceres et al. [97] cryopreserved morulae and blastocysts using slow-cooling protocols and assessed the methylation profile of H3K4me3 by immunostaining, finding no significant differences between cryopreserved and non-cryopreserved controls at the morula and blastocyst stages. By contrast, Maldonado et al. [98] reported lower H3K4me3 methylation levels after slow cooling of bovine blastocysts compared with in vitro controls, as well as increased H3K27me3 methylation compared with non-vitrified in vitro controls using immunostaining. Similarly, Sahraei and colleagues [89] found lower H3K4me3 methylation levels after vitrification of murine eight-cell embryos compared with non-vitrified controls, as well as increased H3K9me2 methylation and decreased H3K9ac acetylation patterns in blastocysts compared with non-vitrified controls, as assessed by ChIP analysis.

Chen et al. [99] found higher methylation levels for H3K4me3 in murine blastocysts after vitrification of eight-cell embryos. Immunofluorescence staining was used to analyse histone methylation marks H3K4me2, H3K4me3, and H3K9me3, and histone acetylation marks H4K12ac and H4K16ac; all epigenetic marks were significantly increased after vitrification compared with non-vitrified controls except for H3K9me3.

Finally, in a study by Truong and Gardner [100], murine blastocysts were vitrified with and without additional antioxidants to assess histone acetylation marks H3K9ac and H3K27ac by immunofluorescence staining. Compared with non-vitrified murine blastocysts, acetylation levels of both marks were significantly reduced after vitrification. Interestingly, supplementation with A3 antioxidants (acetyl-L-carnitine, N-acetyl-L-cysteine, and α-lipoic acid) ameliorated the loss of histone acetylation after vitrification, although acetylation levels still differed from those in non-vitrified controls.

Post-translational histone modifications may therefore be sensitive to cryopreservation of pre-implantation embryos by either vitrification or slow-freezing protocols (overview in Table 4). To date, the most data are available for H3K4me3 methylation, which was altered in many studies, especially when compared with fresh in vivo controls. Overall, cryopreservation of pre-implantation embryos has a possible effect on post-translational histone modifications in humans and animal model systems.

**Table 4:** Post-translational histone modifications. Cryopreservation was performed by vitrification unless stated otherwise. Immunofluorescence (IF). All CPA concentrations are in volume per volume (*v*/*v*) unless otherwise specified; equilibration solution (ES); vitrification solution (VS); ethylene glycol (EG); dimethyl sulfoxide (DMSO); propanediol (PrOH).

Reference	Year	Species	Cryopreserved Cell Type/Cryopreservation Protocol	Analysed Cell Type	Technique	Target	Finding
Bakhtari et al. [59]	2014	Mouse	2-cell embryos ES: 7.5% EG + 7.5% DMSO, 3 min VS: 15% EG + 15% DMSO, 45 s	Blastocysts	IF	Status of histone methylation (H3K4me3) and acetylation (H3K9ac, H4K12ac)	No differences compared with non-vitrified in vitro controls. Increased H4K12ac and decreased H3K4me3 levels compared with in vivo controls.
Chen et al. [99]	2025	Mouse	8-cell embryos ES: 7.5% EG + 7.5% DMSO, 8 min VS: 15% EG + 15% DMSO, 30–60 s	Blastocysts	IF	Status of histone methylation (H3K4me2, H3K4me3, H3K9me3) and acetylation (H4K12ac, H4K16ac)	Vitrification elevated H3K4me2/3, H4K12ac, and H4K16ac levels.
Jahangiri et al. [86]	2014	Mouse	2-cell embryos ES: 7.5% EG + 7.5% DMSO; 7 min VS: 15% EG + 15% DMSO; 60 s	Blastocysts	ChIP assay	Histone profile of H3K9ac, H3K9me2, and H3K4me3 in CTCF site III/IV of imprinted gene *H19* and in promoter region of imprinted *Mest* gene	Histone profiles did not differ after vitrification compared with in vitro controls. Decreased H3K9me2 and increased H3K9ac levels were observed at CTCF sites III/IV of the *H19* gene and in the promoter of the *Mest* gene in non-vitrified and vitrified in vitro blastocysts compared with in vivo controls.
Jahangiri et al. [87]	2018	Mouse	2-cell embryos ES: 7.5% EG + 7.5% DMSO; 7 min VS: 15% EG + 15% DMSO; 60 s	Blastocysts	ChIP assay	Histone profile of H3K9ac, H3K9me2, and H3K4me3 in CTCF site III of imprinted gene *Igf2*	Histone profiles did not differ after vitrification compared with in vitro controls. Decreased H3K9me2 and increased H3K9ac levels were observed at CTCF site III of the *Igf2* gene in non-vitrified and vitrified in vitro blastocysts compared with in vivo controls.
Maldonado et al. [98]	2015	Bovine	Blastocysts (slow cooling) 1.5 M EG; −0.5 °C/min	Blastocysts	IF	Status of histone methylation (H3K4me3 and H3K27me3)	Decreased H3K4me3 methylation and higher H3K27me3 methylation levels compared with non-vitrified in vitro controls.
Sahraei et al. [89]	2018	Mouse	8-cell embryos No details available regarding cryopreservation protocol	Blastocysts	ChIP assay	Histone profiles of H3K9me2, H3K4me3, and H3K9ac	Increased H3K9me2 methylation and partially decreased H3K9ac acetylation and H3K4me3 methylation after vitrification compared with non-vitrified controls.
Souza Cáceres et al. [97]	2016	Bovine	Morulae/blastocysts (slow cooling) 1.5 M EG; −0.6 to −2 °C/min	Morulae/blastocysts	IF	Status of histone H3K4 methylation (H3K4me3)	H3K4me3 histone methylation was not different after cryopreservation compared with non-vitrified controls.
Truong and Gardner [100]	2020	Mouse	Blastocysts EG- and PrOH-based RapidVit Blast protocol	Blastocysts	IF	Status of histone acetylation (H3K9ac and H3K27ac)	Decreased H3K9ac and H3K27ac acetylation levels after vitrification compared with non-vitrified controls.

## 6. Influence of Cryopreservation on Gene Expression of Epigenetic Regulatory Genes

The transcript and protein abundance of epigenetic regulatory genes is crucial for establishing, removing, and maintaining epigenetic marks. This network includes, for instance, de novo DNA methylation mediated by DNMT3A and DNMT3B or indirectly by DNMT3L, maintenance methylation by DNMT1, protection of CpG methylation by TRIM28 and ZNF445, and removal of methylation marks by members of the TET family [44,45,101,102]. For post-translational histone modifications, several HDACs, HATs, and HMTs are required to modify histone residues [46]. Loss of function or reduced abundance of these factors may therefore induce downstream cascades that directly affect DNA methylation, PTMs, and/or epigenetically regulated gene expression.

In a study by Yodrug et al. [84], bovine blastocysts were vitrified, and the expression levels of *DNMT3B* and *HDAC1* were analysed by qRT-PCR; overall, neither expression level differed compared with that of non-vitrified blastocysts. Shida et al. [66] also vitrified bovine eight-cell embryos with different CPAs (DMSO or propylene glycol) and analysed expression levels of the DNA-modifying enzymes DNMT1, DNMT3A, TET1, and TET3 at the eight-cell stage by immunostaining. After vitrification with PG, no differences were found between vitrified and non-vitrified controls; by contrast, increased DNMT1 and TET3 expression and a reduced DNMT3A profile were shown after vitrification with DMSO compared with non-vitrified controls.

Upregulation of different DNA methyltransferases has also been reported in mice and porcine embryos by Hosseini [79], Ma [64], and Jia [103]. In the first study [79], murine two-cell embryos were vitrified, and gene expression levels of *Dnmt1*, *Dnmt3a*, and *Dnmt3b* were analysed in the hippocampus of D2 offspring by qRT-PCR. Increased expression of *Dnmt1*, *Dnmt3a*, and *Dnmt3b* was present after vitrification compared with non-vitrified in vitro and in vivo controls, which is consistent with higher CpG methylation levels of *Meg3* after vitrification compared with in vitro and in vivo controls, as mentioned earlier. Ma et al. [64] also vitrified murine eight-cell embryos and assessed mRNA expression levels of DNA methyltransferases (*Dnmt1*, *Dnmt3a*, and *Dnmt3b*) and TET family enzymes in foetuses and placentas by qRT-PCR. In foetuses, increased expression of *Dnmt1* and *Dnmt3b*, and downregulation of *Tet2* and *Tet3*, were shown after vitrification compared with in vitro and in vivo controls; in placentas, *Dnmt1* was upregulated compared with in vivo and in vitro controls. In this study, gDNA methylation levels and imprinting profiles of *KvDMR1* were also altered after vitrification.

In the study by Jia and colleagues [103], mRNA expression profiles of *DNMT3A* and *DNMT3B* were analysed in porcine two-cell and four-cell embryos, respectively. For *DNMT3A*, there were no differences among groups with or without vitrification; however, expression levels of *DNMT3B* were increased after vitrification of porcine two-cell and four-cell embryos compared with non-vitrified controls.

In contrast to the studies above, Movahed et al. [67] found decreased mRNA expression of *Dnmt3a* and *Dnmt3b* after vitrification compared with non-vitrified in vivo and in vitro controls. Murine two-cell embryos were vitrified, and expression levels were analysed after in vitro culture to the blastocyst stage by qRT-PCR. Notably, global DNA methylation levels were correspondingly reduced after vitrification compared with non-vitrified in vivo and in vitro controls.

Transcript and protein abundance of TET2 was analysed in murine blastocysts after vitrification of eight-cell embryos by Ying and Zhang [62]. Using qRT-PCR and Western blotting, the authors showed that *Tet2* mRNA and TET2 protein levels were decreased after vitrification compared with non-vitrified controls. Interestingly, global DNA methylation levels were increased after vitrification in this study, which may indicate loss of active DNA demethylation by TET enzymes.

One study analysed a large cohort of human epigenetic regulatory genes responsible for DNA methylation (*DNMT* family genes), demethylation (*TET* family genes), and maintenance (*TRIM28* and *ZNF445*) [57]. Zhu and colleagues [57] assessed the effect of mid- and long-term storage (3 vs. 8 years) on vitrified human eight-cell embryos in LN2 using single-cell RNA-seq. mRNA expression levels of demethylation, methylation, and methylation-maintenance genes did not differ after vitrification or mid- and long-term LN2 storage compared with non-vitrified human eight-cell embryos, consistent with comparable global DNA methylation and CpG methylation levels of 22 imprinted genes after vitrification. Only the imprinted gene *DIRAS3* was hypermethylated compared with non-vitrified controls, suggesting that other regulatory effects or direct CpG epimutations could be responsible for this effect.

In summary, as shown by some authors, vitrification may induce increased gene expression of DNA methyltransferases and/or loss of TET abundance, ultimately leading to increased methylation patterns of global DNA or imprinted genes in different cell types or developmental stages (overview in Table 5). However, other authors found no alterations or reported downregulation of DNMTs, including loss of DNA methylation. For PTM-modifying enzymes, only limited data are currently available to reliably assess potential adverse effects. Overall, cryopreservation of pre-implantation embryos has a possible effect on the expression or abundance of epigenetically related enzymes in humans and animal model systems.

**Table 5:** Epigenetic regulatory enzymes. Cryopreservation was performed by vitrification unless stated otherwise. All CPA concentrations are in volume per volume (*v*/*v*); immunofluorescence (IF); equilibration solution (ES); vitrification solution (VS); ethylene glycol (EG); dimethyl sulfoxide (DMSO); propylene glycol (PG).

Reference	Year	Species	Cryopreserved Cell Type/Cryopreservation Protocol	Analysed Cell Type	Technique	Target	Finding
Hosseini et al. [79]	2025	Mouse	2-cell embryos ES: 7.5% EG + 7.5% DMSO; 5 min VS: 15% EG + 15% DMSO; 30 s	Hippocampus of D2 offspring	qRT-PCR	Gene expression levels of DNA methyltransferases *Dnmt1*, *Dnmt3a*, and *Dnmt3b*	Upregulation of *Dnmt1*, *Dnmt3a*, and *Dnmt3b* after vitrification compared with in vivo and non-vitrified controls.
Jia et al. [103]	2020	Porcine	2-cell embryos/4-cell embryos ES: 15%; 3 min VS: 30% EG; 20–30 s	2-cell embryos/ 4-cell embryos	qRT-PCR	Expression profile of DNA methyltransferases *DNMT3A* and *DNMT3B*	Higher expression levels of *DNMT3B* after vitrification of 2-cell and 4-cell embryos compared with non-vitrified controls. No differences for *DNMT3A*.
Ma et al. [64]	2019	Mouse	8-cell embryos ES: 7.5% EG + 7.5% DMSO, 5 min VS: 15% EG + 15% DMSO, 60 s	Foetuses and placentas	qRT-PCR	Expression level of DNA methyltransferases (*DNMT1*, *DNMT3a*, and *DNMT3b*) and *TET* family enzymes	In foetuses, increased expression of *Dnmt1* and *Dnmt3b* and downregulation of *Tet2* and *Tet3* after vitrification compared with in vitro and in vivo controls. In placentas, *Dnmt1* was upregulated compared with in vivo and in vitro controls.
Movahed et al. [67]	2019	Mouse	2-cell embryos ES: 7.5% EG + 7.5% DMSO, 3 min VS: 15% EG + 15% DMSO, 60 s	Blastocysts	qRT-PCR	Expression levels of DNA methyltransferases *Dnmt3a* and *Dnmt3b*	Decreased expression of *Dnmt3a* and *Dnmt3b* after vitrification compared with non-vitrified in vivo and in vitro controls.
Shida et al. [66]	2025	Bovine	8-cell embryos ES: 7.5% EG + 7.5% DMSO or 7.5% PG, 5 min VS: 15% EG + 15% DMSO or 15% PG, 30 s	8-cell embryos	IF	Expression levels of DNA-modifying enzymes (DNMT1, DNMT3A, TET1, and TET3)	Increased DNMT1 and TET3 expression and reduced DNMT3A profile after vitrification with DMSO compared with non-vitrified controls. No differences were found after vitrification using propylene glycol.
Ying and Zhang [62]	2023	Mouse	8-cell embryos Details regarding cryopreservation protocol n.a.	Blastocysts	qRT-PCR and Western blotting	Expression of *Tet2* mRNA and protein abundance	Decreased expression levels of *Tet2* mRNA and TET2 protein after vitrification.
Yodrug et al. [84]	2020	Bovine	Blastocysts ES: 2% or 7.5% EG + 2% or 7.5% DMSO, 3 min VS: 16.5% EG + 16.5% DMSO, 50 s	Blastocysts	qRT-PCR	Expression levels of DNA methyltransferase *DNMT3B* and histone deacetylase 1 (*HDAC1*)	Expression of *HDAC1* and *DNMT3B* did not differ after vitrification compared with non-vitrified controls.
Zhu et al. [57]	2024	Human	8-cell embryos (mid- and long-term storage in LN_2_ for 3 and 8 years vs. non-vitrified controls) ES: 7.5% EG + 7.5% DMSO, 12–15 min VS: 15% EG + 15% DMSO, 45–60 s	8-cell embryos	Single-cell RNA-seq	Gene expression levels of ten DNA demethylation genes (*TET* family), DNA methylation genes (*DNMT* family), and methylation-maintenance genes (*TRIM28*, *ZNF445*)	Expression levels of demethylation, methylation, and methylation-maintenance genes were not different after vitrification compared with non-vitrified controls.

## 7. Influence of Cryopreservation on ncRNAs

It has long been known that non-coding RNAs, such as ribosomal RNAs and transfer RNAs, are crucial for translation and protein biosynthesis. In recent years, the regulatory functions of ncRNAs, which control gene expression either directly or indirectly, have also increasingly come into focus. The exact number of ncRNAs in the human genome remains unknown, but recent bioinformatic data suggest that there are tens of thousands, with some sources indicating more than 100,000 different ncRNAs [104,105,106]. In particular, lncRNAs (>200 nucleotides) and miRNAs (21–23 nucleotides) are associated with the control of gene expression [47,48,104]. However, for the vast majority of these molecules, distinct functions and target genes remain unknown, and alterations of the ncRNA transcriptome after MAR and/or cryopreservation may have long-lasting downstream consequences.

In mice, the ncRNA transcriptome after vitrification of pre-implantation embryos was analysed by Zhu [107], Zhao [108], Movahed [67,88], Heidari [109], and Azizi [110]. Differential expression of lncRNAs was analysed by Zhu et al. [107] in murine placenta after blastocyst vitrification using RNA-seq. Overall, 554 lncRNAs were differentially expressed after vitrification compared with non-vitrified controls, with 227 upregulated and 327 downregulated. Most differentially expressed lncRNAs were validated by qRT-PCR, and KEGG and GO enrichment analyses were used to assess predicted target genes. Interestingly, differentially expressed lncRNAs and their target mRNAs may be related to placental morphogenesis; more specifically, downregulation of the lncRNA Lncenc1 after vitrification could induce higher foetal weight after vitrification. The miRNA transcriptome profile was analysed by Zhao et al. [108] in vitrified murine blastocysts using a miRNA TaqMan assay. Four miRNAs (mmu-miR-199a-5p, mmu-miR-329-3p, mmu-miR-136-5p, and mmu-miR-16-1-3p) were upregulated, and one miRNA (mmu-miR-212-3p) was downregulated compared with non-vitrified controls. Bioinformatic data extraction revealed 1108 predicted target genes, and the differentially expressed miRNAs were mainly involved in implantation processes.

More specifically, expression profiles of the lncRNA gene trap locus 2 (Gtl2) and the non-coding microRNAs miR-29a and miR-29b were analysed by Movahed et al. in murine blastocysts after vitrification of two-cell embryos [67,88]. Expression of the lncRNA Gtl2, which is crucial for early development and growth, was significantly decreased after vitrification compared with in vivo and non-vitrified controls. By contrast, miR-29a and miR-29b were upregulated after vitrification compared with non-vitrified in vivo and in vitro controls. This is of particular interest because *Dnmt3a* and *Dnmt3b* are target genes of miR-29a and miR-29b, and in the latter study, reduced expression of these target genes and a corresponding decline in CpG methylation were also reported, highlighting the complex interactions and far-reaching downstream consequences.

In the study by Heidari [109], the abundance of the non-coding microRNAs miR-16-1 and let-7a was analysed at the blastocyst stage after two-cell embryo vitrification using qRT-PCR, and the authors showed downregulation of both after vitrification compared with in vitro controls. Known target genes of miR-16-1 and let-7a include *Itgb3*, *Dicer*, and *Bcl2*, which are crucial for embryo implantation, cell proliferation, and apoptosis [109]. Azizi and colleagues [110] analysed the expression profiles of four miRNAs (miR-21, let-7a, miR-93, and miR-24) in vitrified eight-cell embryos and blastocysts using qRT-PCR. miR-21 and let-7a expression in eight-cell embryos was decreased compared with non-vitrified controls, whereas miRNA abundance at the blastocyst stage was similar. To assess potential downstream effects in eight-cell embryos, the authors also analysed the expression of putative miRNA target genes, which are mainly involved in development and implantation. However, no differences were present at the cleavage stage.

Cuello et al. [111] analysed the expression profile of embryonic miRNAs in porcine embryos after the use of two different vitrification protocols, SOPS and Cryotop. Irrespective of the vitrification protocol, the miRNA transcriptome was dysregulated after cryopreservation: after SOPS vitrification, 94 miRNAs were differentially expressed compared with non-vitrified controls, with one upregulated and 93 downregulated; after Cryotop vitrification, 174 miRNAs were differentially expressed, with one upregulated and 173 downregulated. miRNA transcriptome microarray analysis was used to assess abundance and GO-term analysis was used for data mining. The differentially expressed miRNAs identified were mainly related to proliferation, apoptosis, and cell stress.

Li [112], Hiura [77], and Daneshvar [113] analysed lncRNAs and miRNAs after cryopreservation of pre-implantation embryos in humans. Microarray analysis and quantification of non-coding microRNAs were performed by Hiura and colleagues [77] in placentas after FET, fresh embryo transfer, and natural conception. A total of 39 miRNAs were differentially expressed after FET compared with fresh embryo transfer or natural conception, and eighteen miRNAs were located in three imprinted regions (C19MC, C14MC, and *IGF2*). GO-term and KEGG analyses of potential target genes showed associations with growth, development, cell migration, and type II diabetes mellitus.

Overall expression profiles of lncRNAs in human eight-cell embryos were assessed by Li et al. [112], who vitrified eight-cell embryos and stored them in LN2 for 3 or 8 years. Overall, 365 lncRNAs were differentially expressed after vitrification compared with non-vitrified controls, whereas no differences were found between mid- and long-term storage in LN2. Putative target genes were mainly related to metabolism, stress response, apoptosis, the cell cycle, and cell adhesion.

Similarly to the studies by Azizi et al. [110] and Heidari [109] in mice, Daneshvar [113] analysed the expression profiles of two small non-coding RNAs, miR-16 and let-7a, which control post-transcriptional gene expression, embryo implantation, and cell proliferation in humans. Blastocysts were analysed after vitrification or revitrification and compared with non-vitrified controls using qRT-PCR. let-7a was downregulated after vitrification or revitrification compared with non-vitrified in vitro controls, and expression levels of miR-16 were not different after vitrification compared with non-vitrified controls but were significantly lower after double revitrification compared with non-vitrified in vitro controls. To assess downstream effects, expression of related miRNA target genes was analysed: expression of *ITGB3* and *BCL2* was significantly increased after single and double vitrification, whereas *BAX* expression was increased after revitrification compared with fresh in vitro controls.

In summary, alterations of miRNAs and lncRNAs were found in several studies in humans and animals (overview in Table 6), and bioinformatic analyses revealed numerous putative or confirmed target genes involved in early development, growth, cell differentiation, and implantation. Thus, alterations of ncRNAs after cryopreservation may become a key element of induced epimutations, with several downstream short-, mid-, or long-term adverse effects. Overall, cryopreservation of pre-implantation embryos has a possible effect on the expression or abundance of miRNAs and lncRNAs in humans and animal model systems.

**Table 6:** Expression levels of ncRNA. Cryopreservation was performed by vitrification unless stated otherwise. All CPA concentrations are in volume per volume (*v*/*v*); equilibration solution (ES); vitrification solution (VS); ethylene glycol (EG); dimethyl sulfoxide (DMSO).

Reference	Year	Species	Cryopreserved Cell Type/Cryopreservation Protocol	Analysed Cell Type	Technique	Target	Finding
Azizi et al. [110]	2021	Mouse	8-cell embryos/blastocysts ES: 7.5% EG + 7.5% DMSO, 10 min VS: 15% EG + 15% DMSO, 45–60 s	8-cell embryos/blastocysts	qRT-PCR	Expression profile of four miRNAs (miR-21, let-7a, miR-93, and miR-24)	Decreased miR-21 and let-7a miRNA expression after vitrification in 8-cell embryos compared with non-vitrified controls.
Cuello et al. [111]	2024	Porcine	Blastocysts (with SOPS and Cryotop vitrification systems) ES: 7.5% EG + 7.5% DMSO, 3 min VS: 16% EG + 16% DMSO, 60 s	Blastocysts	miRNA transcriptome microarray	Expression profile of embryonic miRNAs	After SOPS vitrification, 94 miRNAs were differentially expressed, with one upregulated and 93 downregulated. After Cryotop vitrification, 174 miRNAs were differentially expressed, with one upregulated and 173 downregulated compared with non-vitrified controls. The identified miRNAs were mainly related to proliferation, apoptosis, and cell stress.
Daneshvar et al. [113]	2021	Human	Blastocysts ES: 7.5% EG + 7.5% DMSO, 15–20 min VS: 15% EG + 15% DMSO, 60 s	Blastocysts	qRT-PCR	Expression profile of two small non-coding RNAs controlling post-transcriptional gene expression (miR-16 and let-7a)	Downregulation of let-7a after vitrification or revitrification compared with non-vitrified in vitro controls. Expression levels of miR-16 were not different after vitrification compared with non-vitrified controls but were significantly lower after double revitrification compared with non-vitrified in vitro controls.
Heidari et al. [109]	2019	Mouse	2-cell embryos ES: 7.5% EG + 7.5% DMSO VS: 15% EG + 15% DMSO	Blastocysts	qRT-PCR	Expression levels of non-coding microRNAs miR-16-1 and let-7a	Downregulation of miR-16-1 and let-7a after vitrification compared with fresh embryos.
Hiura et al. [77]	2017	Human	FET vs. fresh embryo transfer vs. natural conception ES: 7.5% EG + 7.5% DMSO VS: 15% EG + 15% DMSO	Placenta	miRNA microarray analysis and qRT-PCR	Microarray analysis and quantification of non-coding microRNAs	A total of 39 miRNAs were differentially expressed after frozen embryo transfer compared with fresh embryo transfer or natural conception. Eighteen were located in three imprinted regions (C19MC, C14MC, IGF2).
Li et al. [112]	2022	Human	8-cell embryos (mid- and long-term storage in LN_2_ for 3 and 8 years vs. non-vitrified controls) ES: 7.5% EG + 7.5% DMSO, 12–15 min VS: 15% EG + 15% DMSO, 45–60 s	8-cell embryos	Single-cell RNA-seq	Overall expression profiles of lncRNAs	365 lncRNAs were differentially expressed after vitrification compared with non-vitrified controls, but the differences were moderate.
Movahed et al. [88]	2020	Mouse	2-cell embryos ES: 7.5% EG + 7.5% DMSO; 3 min VS: 15% EG + 15% DMSO; <60 s	Blastocysts	qRT-PCR	Expressions of long non-coding RNA (lncRNA) gene trap locus 2 (Gtl2)	Decreased expression of lncRNA Gtl2 after vitrification compared with in vivo and non-vitrified controls.
Movahed et al. [67]	2019	Mouse	2-cell embryos ES: 7.5% EG + 7.5% DMSO, 3 min VS: 15% EG + 15% DMSO, 60 s	Blastocysts	qRT-PCR	Expression levels of non-coding microRNAs miR-29a and miR-29b	Upregulation of miR-29a and miR-29b after vitrification compared with non-vitrified in vivo and in vitro controls.
Zhao et al. [108]	2015	Mouse	Blastocysts ES: 10% EG + 10% DMSO, 30 s VS: 15% EG + 15% DMSO, 25 s	Blastocysts	miRNA TaqMan assay	miRNA transcriptome profile	Four miRNAs (mmu-miR-199a-5p, mmu-miR-329-3p, mmu-miR-136-5p, mmu-miR-16-1-3p) were upregulated, and one miRNA (mmu-miR-212-3p) was downregulated. Differentially expressed miRNAs were mainly involved in implantation processes.
Zhu et al. [107]	2022	Mouse	Blastocysts ES: 7.5% EG + 7.5% DMSO, 10–12 min VS: 15% EG + 15% DMSO, 60 s	Placenta	RNA-seq analysis	Expression profile of lncRNAs	554 lncRNAs were differentially expressed after vitrification compared with non-vitrified controls, with 227 upregulated and 327 downregulated. Downregulation of lncRNA Lncenc1 may induce higher foetal weight after vitrification.

## 8. Conclusions and Implications for Clinical MAR

Several studies have shown that the epigenome is sensitive to the cryopreservation of pre-implantation embryos in humans and animals, although some authors found no adverse effects. The contradictory results may be due to differences in species, cell types and stages, analytical techniques, and cryopreservation protocols. Cryopreservation can affect global DNA and imprinted gene methylation, upstream post-translational histone modifications, downstream gene expression, and the epigenetically related enzymatic network (see Supplementary Figure S1). To date, little is known about non-coding RNAs, such as lncRNAs and miRNAs. However, altered ncRNA expression levels have been found in many studies, potentially indicating that ncRNA transcript levels are particularly vulnerable to cryopreservation. To complicate matters further, the exact pathways or target genes of the affected ncRNAs remain unknown for most ncRNAs, necessitating large-scale analyses and in-depth bioinformatic data mining in future work.

To date, vitrification is the most common method for cryopreservation of MII oocytes, zygotes, and pre-implantation embryos because cryopreservation protocols have been optimised to achieve higher survival and pregnancy rates over recent years. Today, commercial vitrification protocols for humans consist of a two-step procedure in which lower concentrations of cryoprotectants are used during the equilibration phase (7.5% EG and 7.5% DMSO/PrOH), and higher CPA concentrations (15% EG and 15% DMSO/PrOH) are employed immediately prior to vitrification, with minor adjustments made regarding exposure times and temperature. Nevertheless, continuous optimisation has taken place, and various additives can be utilised to mitigate potential damage caused by cryopreservation—such as sugars, antifreeze proteins, and antioxidants. Whether, and to what extent, these intervention strategies also reduce potential epigenetic alterations resulting from cryopreservation, particularly in humans, remains largely unclear. In mice, supplementation with A3 antioxidants (acetyl-L-carnitine, N-acetyl-L-cysteine, and α-lipoic acid) during vitrification or thawing attenuated changes in histone acetylation following cryopreservation of murine blastocysts [100]. Similar effects have been reported for resveratrol as an antioxidant in vitrified murine MII oocytes [114]. After vitrification of porcine embryos, supplementation with different antioxidants, iron oxide nanoparticles, or antifreeze protein I ameliorated alterations of the gene expression of *BAX*, *BCL2*, *OCT4*, and *SOX2* [115]. Glutathione ethyl ester, when used as an additive, also enhanced the cryotolerance of vitrified murine MII oocytes and promoted their subsequent development by protecting intracellular redox homeostasis [116]. Different strategies to reduce (epigenetic) damage from cryopreservation are therefore of great interest and must be considered in further studies—in particular with regard to the epigenetic profile since even slight changes in cryopreservation protocols can have an important influence (in a positive or, in the worst case, even in a negative direction) on the epigenome. It should be noted that assessment can sometimes be difficult, as the composition of commercial vitrification systems is often not known in detail.

However, a paradigm shift is currently occurring in many MAR laboratories with respect to new ultra-rapid vitrification and warming protocols [117,118,119,120]. Conventional protocols take up to 30 min for vitrification and warming, whereas new ultra-fast protocols reduce the amount of time to few minutes. Although clinical MAR outcomes are reliable, there are currently no data on the influence of ultra-rapid vitrification protocols on the epigenome, which must therefore be considered in future work.

The interpretation of epimutations is highly complex because alterations can, but do not necessarily, lead to upstream or downstream changes that subsequently influence the resulting genotype or phenotype. Even minor alterations have the potential to induce detrimental long-term consequences in offspring, consistent with potential cascading effects, whereby even small alterations on the molecular level may have substantial future consequences on the phenotype or, conversely, no consequences at all. It is also noteworthy that MAR treatment, even without cryopreservation, induces epimutations at different levels [27,68,69].

Although cryopreservation is regarded as safe, potential epimutations must be taken into account, especially because some studies in humans have reported an increased risk of rare genomic imprinting disorders in children conceived by MAR techniques [18,22,23,26]. Moreover, long-term follow-up data from human clinical cohorts has also indicated some epigenetic-related disorders after frozen embryo transfer, although many of the identified risk associations were not present after adjustment [18]. It remains particularly unclear whether these minor increases in adverse outcomes are related to MAR itself or reflect intrinsic factors in the subfertile patient population undergoing medically assisted reproduction. So far, with respect to clinical implications, the American Society for Reproductive Medicine (ASRM) and the European Society of Human Reproduction and Embryology (ESHRE) consider vitrification as the standard of care for embryo cryopreservation [121,122,123]. However, epimutations induced by cryopreservation of pre-implantation embryos cannot be excluded. To make a final assessment even more complex, many concerns regarding epimutations have been raised from animal studies in mice or bovines, and differences between species in terms of lineage differentiation, transcription, and epigenetic mechanisms are well known and can ultimately lead to misinterpretation or incorrect translation between animal model systems and humans. Additionally, slight changes in cryopreservation protocols or even different culture media, in vitro culture strategies (sequential or continuous embryo culture), and culture conditions (low or high oxygen) in the MAR laboratory itself have the potential to affect the epigenetic landscape with or without cryopreservation and must be systematically considered. Thus, further research is clearly necessary to assess safety and improve efficiency especially in human MAR applications. Future research should therefore encompass prospective human cohort epigenetic monitoring, including novel low-toxicity cryoprotector development based on sensitive epigenetic biomarkers.

## Data Availability

No new data were created or analysed in this study.

## Supplementary Materials

The following supporting information can be downloaded at: https://www.mdpi.com/article/10.3390/cells15121049/s1, Figure S1. Schematic overview of potential effects of cryopreservation and warming/thawing on the epigenome of pre-implantation embryos. Cryopreservation can directly or indirectly affect the epigenetic profile by alterations of the epigenetic regulatory network (including regulatory enzymes, PTMs, ncRNAs) or DNA methylation itself (including global DNA or TE methylation or methylation of imprinted or pluripotency genes). Alterations might induce ultimately gene expression or repression.

## Abbreviations

The following abbreviations are used in this manuscript:

MAR	medically assisted reproduction
IVF	in vitro fertilisation
ICSI	intracytoplasmic sperm injection
FET	frozen embryo transfer
CPA	cryoprotective agent
EG	ethylene glycol
PG	propylene glycol
PrOH	propanediol
DMSO	dimethyl sulfoxide
BWS	Beckwith–Wiedemann syndrome
SRS	Silver–Russell syndrome
PWS	Prader–Willi syndrome
PTM	post-translational histone modification
ncRNA	non-coding RNA
DNMT	DNA methyltransferase
Me	methylation
Ac	acetylation
Ub	ubiquitination
HAT	histone acetyltransferase
HDAC	histone deacetylase
HMT	histone methyltransferase
HDM	histone demethylase
RISC	RNA-induced silencing complex
CpG	cytosine–phosphate–guanine dinucleotide
gDNA	global DNA methylation
DMR	differentially methylated region
ICR	imprinted control region
PGC	primordial germ cell
COS	controlled ovarian stimulation
IVC	in vitro culture
ET	embryo transfer
lncRNA	long non-coding RNA
miRNA	microRNA
WGBS	whole-genome bisulphite sequencing
LUMA	luminometric methylation assay
ChIP	chromatin immunoprecipitation
IF	immunofluorescence
