# Supplementary figures and images for: Influence of Cryopreservation of Pre-Implantation Embryos on the Epigenome
Source: Cells. 2026 Jun 8;15(12):1049. doi: 10.3390/cells15121049 (PMC13297363; doi:10.3390/cells15121049)

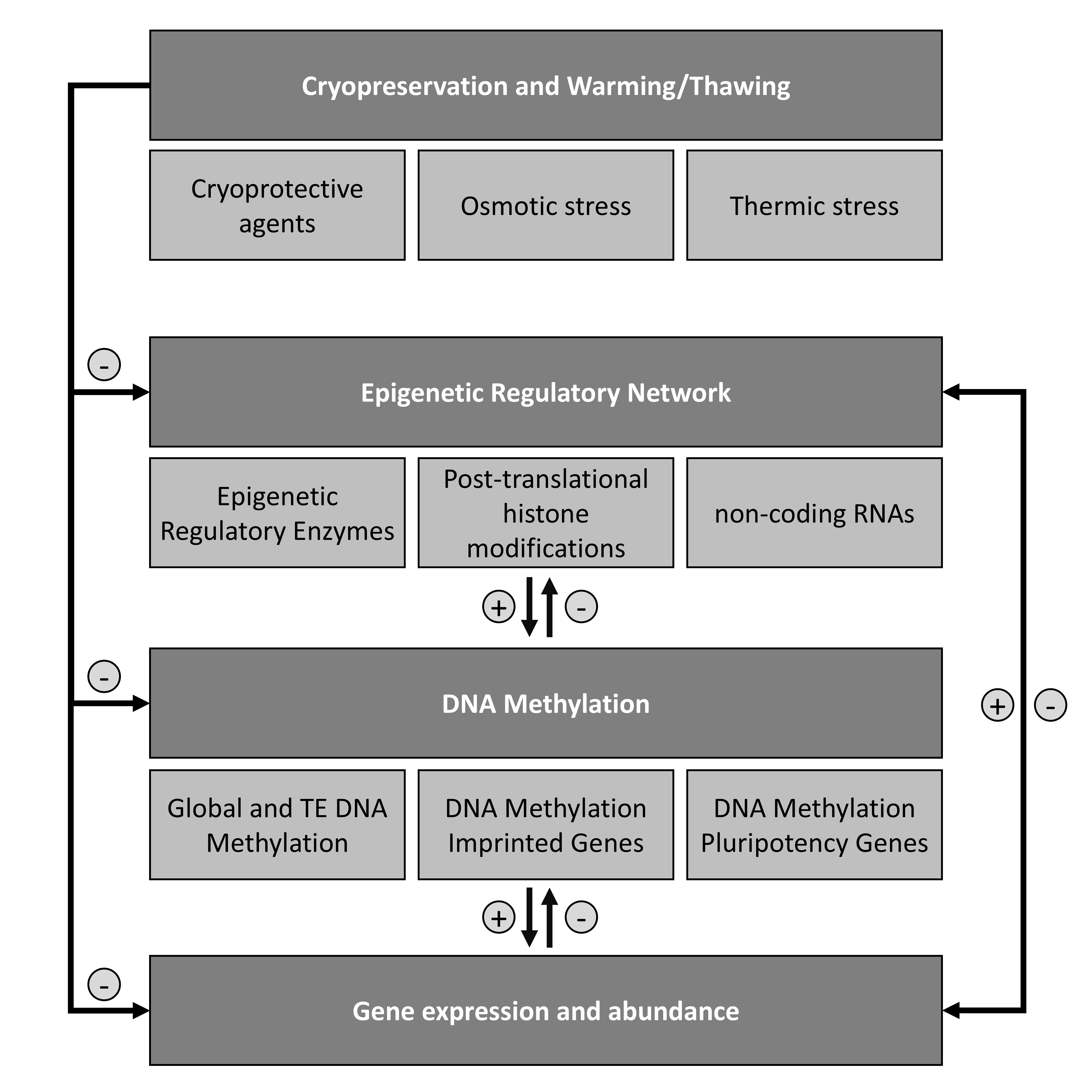

Supplement: Supplementary file 1 [file cells-15-01049-s001.zip › cells-4338173-supplementary materials.png]
